# Full Regio- and Stereoselective Protocol for the Synthesis of New Nicotinoids via Cycloaddition Processes with the Participation of Trans-Substituted Nitroethenes: Comprehensive Experimental and MEDT Study

**DOI:** 10.3390/molecules28083535

**Published:** 2023-04-17

**Authors:** Jowita Kras, Przemysław Woliński, Roman Nagatsky, Oleg M. Demchuk, Radomir Jasiński

**Affiliations:** 1 Department of Organic Chemistry and Technology, Cracow University of Technology, Warszawska 24, 31-155 Krakow, Poland; 2 Faculty of Medicine, The John Paul II Catholic University of Lublin, Konstantynow 1J, 20-708 Lublin, Poland

**Keywords:** [3 + 2] cycloaddition, nitrones, nitroalkenes, nicotinoids, molecular electron density theory

## Abstract

[3 + 2] Cycloaddition reactions with the participation of *Z*-C-(3-pyridyl)-*N*-methylnitrone and series of *E*-2-R-nitroethenes were both experimentally and theoretically explored in the framework of Molecular Electron Density Theory. It was found that all considered processes are realized under mild conditions and in full regio- and stereocontrol. The ELF analysis additionally showed that the studied reaction proceeds by a two-stage, one-step mechanism.

## 1. Introduction

**Scheme 1:**
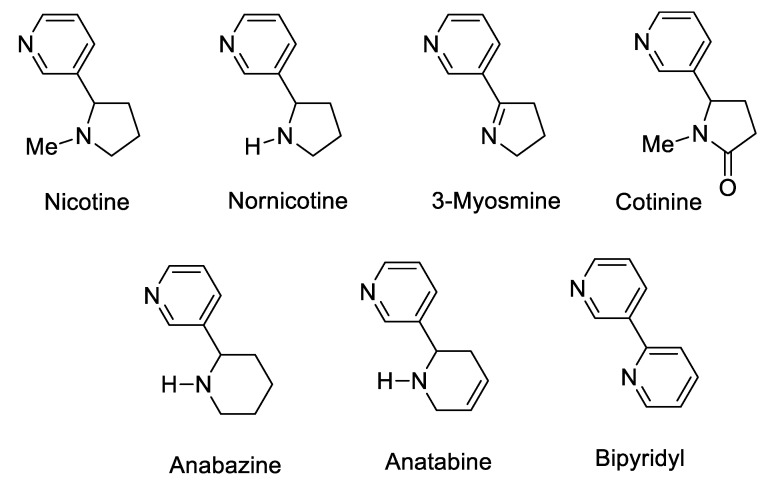
Nicotine and its selected analogs (nicotinoids).

Nicotine (1-methyl-2-(3-pyridyl)-pyrrolydine) is a heterocyclic type alkaloid. This compound is a colorless, odorless, and oily liquid in terms of physicochemical properties, which easily undergoes oxidation, and as a result of which it browns over time [1]. It is characterized by a sharp, scorching smell. Nicotine is a bioactive substance that affects acetylcholine receptors (nAChR), and it is involved in many functions of the central nervous system (OUN). Such action has a huge impact on the human body because it increases the synthesis of endogenous neurotransmitters, which enhances the feeling of pleasure and affects the improvement of memory and executive functions [2,3,4]. It is worth mentioning that many bioactive analogs of nicotine are known (Scheme 1).

At present, the question of the preparation of new analogs of nicotine is the subject of research of many scientific groups. Our current work is a part of this trend. We decided to examine the course of the [3 + 2] cycloaddition (32CA) reaction with the participation of *Z*-C-(3-pyridyl)-*N*-methylnitrone (**1**) and the series of *E*-2-R-nitroethenes (**2a**–**c**) substituted with methyl and trihalomethyl groups. In our opinion, this approach presents a chance for an easy, effective, and selective preparation of target compounds, which should exhibit the real nature of nicotinoids. It should be underlined that the presence of the nitro group in the adducts widens the range of potential directions of further functionalization [5,6,7].

## 2. Results

**Scheme 2:**
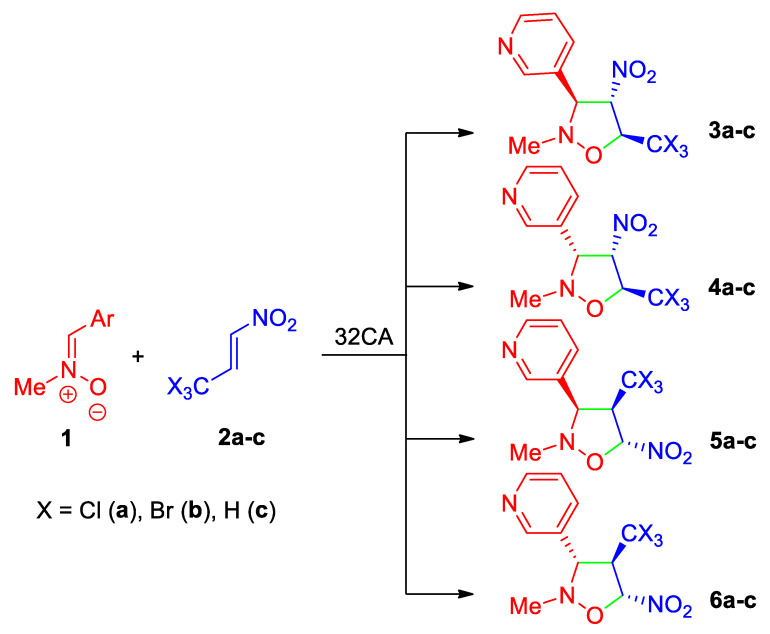
Theoretically possible channels of 32CAs of *Z*-C-(3-pyridyl)-*N*-methylnitrone (**1**) with *E*-2-R-nitroethenes (**2a**–**c**).

[3 + 2] cycloaddition reaction with the participation of *Z*-C-(3-pyridyl)-*N*-methylnitrone (**1**) and *E*-2-R-nitroethenes (**2a**–**c**) can theoretically lead to a mixture of four regio- and stereoisomeric cycloadducts (Scheme 2). In the first stage of our research, we decided to identify the real reaction course. For these considerations, we started with the model reaction involving *E*-1-nitroprop-1-ene (**2a**). It was found that in the benzene solution, the analyzed reaction easily proceeds at r.t using a 1:2 molar ratio of reagents 1 and 2. The reaction progress was monitored using TLC and HPLC techniques. In this way, we established that the conversion of nitrone ended after 24 h. The post-reaction mixture was evaporated to dryness. The analysis of the residue only showed the presence of one reaction product, which was isolated by simple crystallization from ethanol. Its constitution was examined on the basis of the data of the spectral analysis. The HPLC–MS studies confirmed that the compounds had the proper molecular weights and, in all cases, expected protonated pseudo-molecular ions [M + H]^+^ were observed as only intensive signals in MS spectra. Notably, the compound possessing the Cl heteroatom had a very specific isotopic pattern, which was identical to that theoretically calculated under the consideration of the numbers of those atoms in the molecule (*m*/*z*: 325.9866 (100.0%), 327.9836 (95.9%), 329.9807 (30.6%), 326.9900 (10.8%), 328.9870 (10.4%), 330.9841 (3.3%), 331.9777 (3.3%), 326.9836 (1.1%), 328.9807 (1.1%)). The combination of the observed pseudo-molecular ion of proper value *m*/*z* and isotopic pattern unambiguously proved the molecular formulae of the new compound. On the recorded MS spectra, there was no fragmentation and the structures of studied compounds, which, therefore, were proved by NMR.

In particular, on the ^1^HNMR spectrum, three types of signals were detected. These signals are connected with the existence of (a) methyl group (2.74 ppm), (b) azolidine ring protons (4.19–5.54 ppm), and (c) pyridine ring protons (7.33–8.65 ppm). Most important for the determination of the chemical structure are azolidine ring protons, which create the AMX spin system. In particular, in a relatively weaker field, the doublet connected with the H3 proton was detected. Signals from H4 and H5 protons (doublet of doublets and doublet respectively) exist in a slightly stronger field. The chemical shift and multiplicity of the H4 signal confirms, without any doubt, that the nitro group is located on the C4 carbon atom of the heterocyclic ring. Then, values of H3–H4 and H4–H5 coupling constants clearly indicate the stereochemistry of the analyzed structure. In particular, the J = 4.51 Hz confirms the trans-relation of protons H4 and H5, whereas J = 8.07 Hz confirms the cis-relation of protons H3 and H4.

For the full characterization of the isolated compound, we also registered its ^13^CNMR spectrum (see Supplementary Material). On this spectrum, respective signals connected with all expected carbon atoms were detected. In conclusion, for the isolated adduct, the constitution of 3,4-*cis*-4,5-*trans*-2-methyl-3-(3-pyridyl)-4-nitro-5-trichloromethylisoxazolidine (**4a**) should be assigned. In a similar way, we examined 32CAs with the participation of *E*-3,3,3-tribromo-1-nitroprop-1-ene (**2b**) and *E*-1-nitroprop-1-ene (**2c**). It was found that the selectivity of the mentioned reactions is identical to the case of 32CA **1** + **2a**. In both cases, respective 3,4-*cis*-4,5-*trans*-2-methyl-3-(3-pyridyl)-4-nitro-5-trihalomethylisoxazolidine was detected in the postreaction mixture as a single product. Therefore, the described process can be treated, in some respects, as a general methodology for the preparation of oxygen-containing nitronicotinoids.

In the second stage of our research, we decided to shed a light on the molecular mechanism of the formation of detected cycloadducts. It is important because the latest discoveries in the research of the cycloaddition reaction mechanism fully undermined the definition of the “concerted” 32CA mechanism [8,9] with the “pericyclic” reorganization of electron density within the transition state. At this moment, it is evident that within the course of the cycloaddition process, many stages with different types of electron flux are possible [10,11]. The full description of the electron reorganization requires a detailed Molecular Electron Density Theory (MEDT) study based on Electron Localization Function (ELF) techniques. In this study, we carried out our investigation in relation to the **1** + **2a** → **4a** model process. A similar analysis was successfully performed for several different types of bimolecular processes [12,13,14].

**Figure 1:**
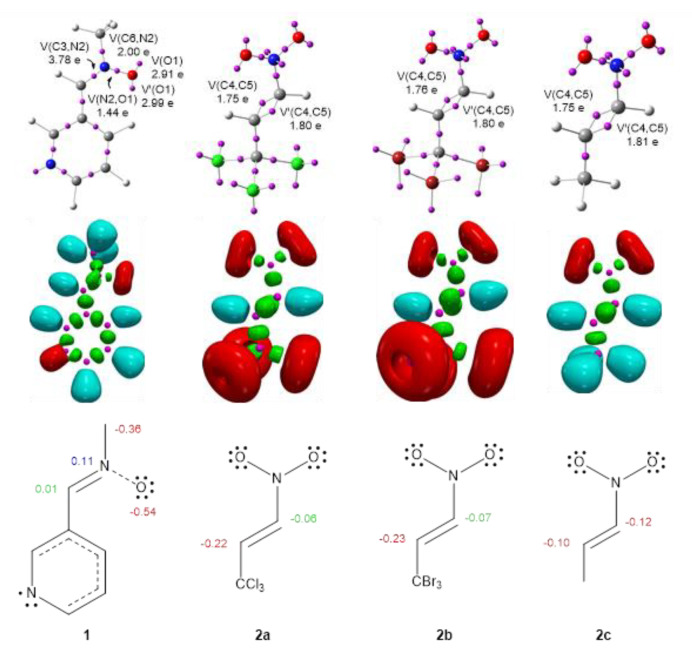
ωB97X-D/6-311G(d) ELF basin attractor positions together with the most relevant valence basin populations, ELF localization domains represented at an isosurface value of ELF = 0.75, and ELF-based Lewis-like structures together with natural atomic charges, for 1-nitropropenes **2a**–**c** and nitrone **1**. Valence basin populations and natural atomic charges are given as the average number of electrons, e. Positive charges are colored blue, neutral green, and negative red.

The topological analysis of the ELF allows a simple connection between the electron density distribution and the chemical structure [15]. Therefore, in order to characterize the electronic structure of the 1-nitropropene **2c**, its halogenated derivatives **2a** and **2b**, and the nitrone **2**, a topological analysis of the ELF was conducted. ELF basins and the most relevant valence basins are shown in Figure 1, where monosynaptic basins, labeled V(A), are related to nonbonding regions, whereas disynaptic basins V(A,B) denote bonding regions between atoms A and B.

Topological analysis of the ELF of nitrone **1** shows the presence of one disynaptic basin V(C,N), integrating 3.78 e, and one disynaptic basin V(N,O), only integrating 1.44 e, representing bonding regions of C=N and N-O bonds, respectively. The two monosynaptic basins V(O) and V’(O), integrating a total of 5.92 e, represent the nonbonding electron density of O1. At 1-nitropropenes **2a**–**c**, two disynaptic basins V(C,C) and V’(C,C), gathering a total population of 3.55 e, are observed.

The ELF topology of nitrone **1** indicates a highly polarized, underpopulated, N-O bond with electron density concentrated at the oxygen. The lack of pseudoradical or carbenoid centers suggests a zwitterionic-type of reactivity of the used three-atom-component (TAC). Furthermore, 1-Nitropropenes **2a**–**c** show no change of electron density at the ethylene C=C double bond, independently of halogenation of the methyl group.

Then, the charge distribution of these molecules was analyzed by Natural Population Analysis (NPA) [16,17]. NPA of the nitrone **1** shows negatively charged oxygen and carbon of the methyl group, by −0.54 and −0.36 e, respectively. The nitrogen of the nitrone group has a slight positive charge of 0.11 e, and the carbon atom is almost neutral. In the 1-nitropropene series **2a**–**c**, a reversal of polarity at the ethylene double bond can be observed in response to the halogenation of the methyl group. The nitro group substituted carbon of **2c** exhibits a negative charge of −0.12 e, while the other vinylic carbon is charged by −0.10 e. The charge distribution at **2a** and **2b** is reversed, showing a charge of −0.22 and −0.23 e, respectively, at the carbon closer to the trihalogenated methyl group, with the other one having a charge of only −0.06 and −0.07 e, respectively.

### 2.1. Analysis of the Conceptual DFT Reactivity Descriptors

To provide a quantitative view of reactivity in terms of classical concepts, such as nucleophilicity and electrophilicity, the Conceptual Density Functional Theory (CDFT) [16,17,18] indices were calculated. The global reactivity indices have been proven to be a powerful tool to describe the reactivity in many studies of polar reactions [19,20,21,22,23] The calculated values for 1-nitropropene **2c**, its halogenated derivatives **2a** and **2b**, and nitrone **1** are gathered in Table 1.

The electronic chemical potential μ of 1-nitropropenes **2a**–**c** exhibits a much lower value than the nitrone **1**, from −5.00 eV to −5.74 eV and −3.65 eV, respectively, indicating that, in a polar process, the electron density transfer will take place from the nitrone **1** to 1-nitropropenes. Polar reactions, where the electron density flows from the TAC to ethylene, can be described as the forward electron density flux (FEDF) [24,25]; in other words, where the TAC acts as the nucleophile.

The chemical hardness η indicates the resistance of the molecule to exchange electron density with the environment. The 1-nitropropenes **2a**–**c** show relatively high hardness in the range of 5.03–5.59 eV, whereas the nitrone **1** presents a lower value of 4.33 eV.

The electrophilicity ω and nucleophilicity N of **1** are 1.53 and 3.29 eV, respectively, placing it among strong electrophiles and strong nucleophiles [26]. On the other hand, all of the considered 1-nitropopenes are classified as marginal nucleophiles and moderate electrophiles, except **2a**, being a marginal electrophile. The halogenation of 1-nitropropene at the methyl group drastically increases the electrophilicity in H (2.33 eV) < Br (3.00 eV) < Cl (3.07 eV) series and decreases nucleophilicity in a reverse order.

The Parr functions [27], as have been shown in many studies [28], are one of the most accurate tools for predicting the local reactivity of molecules in polar reactions. In order to try and predict the most favorable interaction in the studied systems, the Parr functions were analyzed.

**Figure 2:**
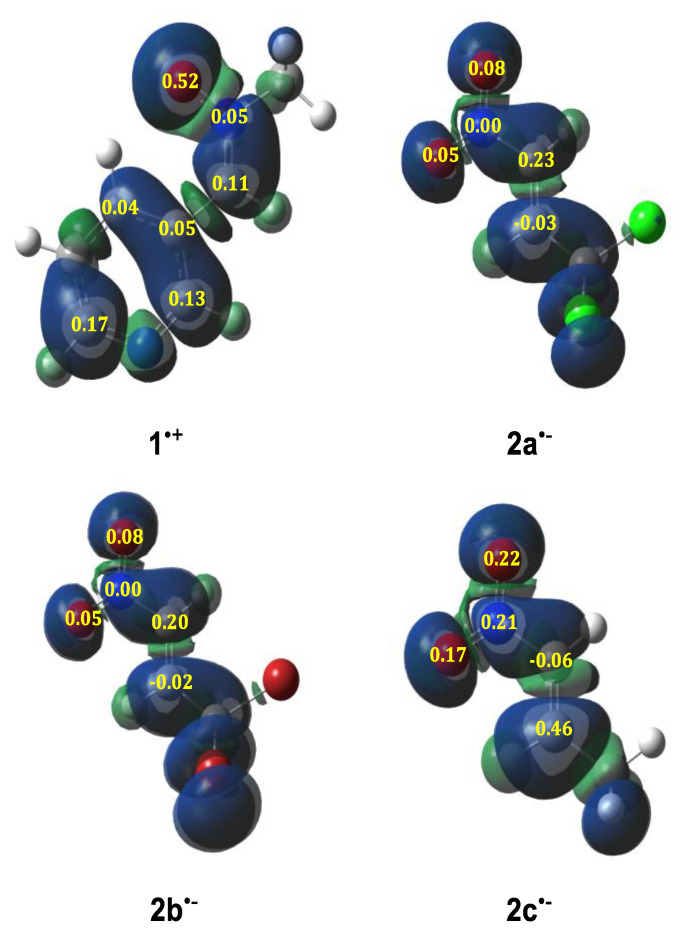
The Mulliken atomic spin densities (ASD) 3D representations of the radical cation of the nitrone **1**, and the radical anions of the 1-nitropropenes **2a**–**c**, and the corresponding nucleophilic and electrophilic Parr functions.

The local nucleophilic P_k_^−^ Parr function in the nitrone **1** exhibits the highest value, 0.52, on the oxygen atom (Figure 2). On the other hand, the most electrophilic center is located at the beta-position of the nitrovinyl moiety. Therefore, the local interactions favor the formation of 4-nitroisoxazolidines. This correlates with the experimentally observed regioselectivity. It should be noted at this point that the cis/trans diastereoselectivity is determined by stabilizing secondary orbital interactions (SOI) between p_z_ orbitals at the aryl ring and at the nitrogroup. This favors the formation of 3,4-*cis* configuration in the final adduct. An identical effect was recently detected in the case of many examples of the 32CAs between aryl-substituted TACs and conjugated nitroalkenes [29,30,31,32].

### 2.2. BET Mechanistic Investigations

In order to determine full characterization of the molecular mechanism of [3 + 2] cycloaddition reaction of C-3-pyridino-N-methyl nitrone **1** with halogenated 1-nitroprop-2-enes, a bonding evolution theory (BET) study of the bonding changes along the experimentally favorable reaction path involving 3,3,3-trichloro derivative **2b** was performed. Detailed BET data of the relevant points of the reaction are given in Table S1 of the Supplementary Information. These points were chosen because of a change in electronic structure compared to the previous point; a basin disappears or is created.

**Scheme 3:**
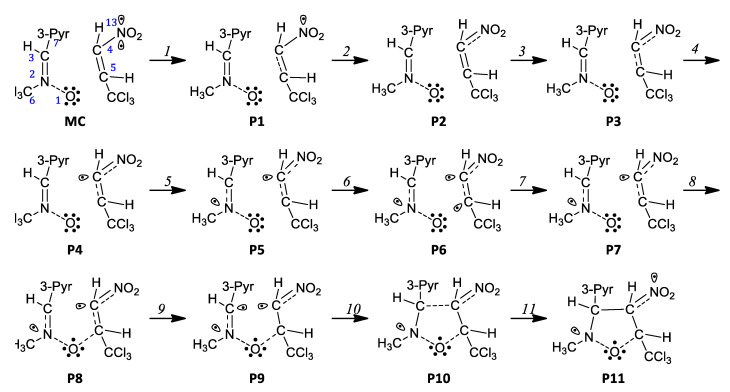
Simplified representation of the molecular mechanism of the 32CA reaction involving C-3-pyridino-N-methyl nitrone **1** with 3,3,3-trichloro-1-nitroprop-2-ene **2b** by Lewis-like structures derived from the topological analysis of the ELF along the reaction path.

**Figure 3:**
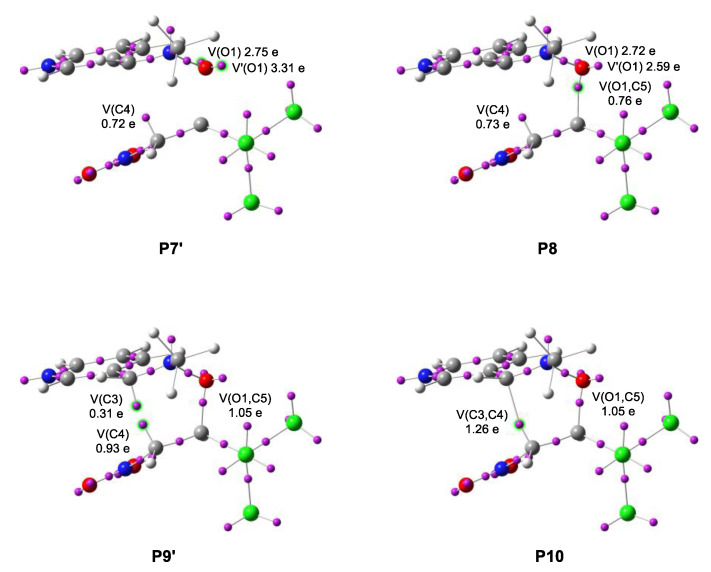
ELF attractor positions of the structures involved in the C-O and C-C bond formation along the experimental reaction path of 3,3,3-trichloro-1-nitropropene **2a** and nitrone **1**. The prime symbol marks the last structure of the corresponding BET phase.

The BET study of 344 points along the reaction path revealed twelve phases, during which the following observations could be made (Scheme 3, Figure 3):

1. From phase I to phase IV, the topological changes leading to the creation of the pseudoradical center [33] on C10 are taking place. In structures P1 and P2, the V(N13) and V’(N13) monosynaptic basins respectively vanish, increasing the population in the V(C4,N13) disynaptic basin. Then, the V’(C4,C5) disynaptic basin of the double bond merges with V(C4,C5), increasing its integration in P3. At the same time, the N2-O1 bond becomes depopulated, transferring 0.12 e to the V(C3,N2) disynaptic basin. These changes contribute most to the energetic cost, 11.0 kcal∙mol^−1^, which is only 1.1 kcal∙mol^−1^ lower than the TS (coincidentally being the P7 structure);
2. At the start of phase V, the pseudoradical center at C4 is created from the electron density of the C4-C5 bond, with an initial population of 0.44 e, after which the V(C4,C5) disynaptic basin is further depopulated, increasing the V(C4) monosynaptic basins integration;
3. Then, the V(N2) monosynaptic basin representing nonbonding electron density is created at P5 with an initial population of 0.90 e originating from the V(C3,N2) basin. The V(N2) starts with only 39% of its final population, to which the V(C3,N2) disynaptic basin contributes further in the leading phases, becoming an underpopulated double bond;
4. At P6, the V(C5) monosynaptic basin is created from the population of disynaptic basin V(C4,C5), only integrating 0.07 e for a duration of a short phase VII, after which it disappears. At P8, a new bond is created, at an O-C distance of 1.658 Å, by the donation of nonbonding electron density of O1. The new V(O1,C5) disynaptic basin starts with a population of 0.76 e, ultimately increasing to 1.29 e at the cost of the nonbonding electron density of O1 and the V(N2,O1) disynaptic basins integration. The N2-O1 single bond becomes strongly underpopulated with its final integration of 0.94 e;
5. Phase X starts with the creation of a pseudoradical center at C3 with a population of 0.12 e originating from the disynaptic basin V(C3,N2). Then, at P10, the C3-C4 single bond is created by the coupling of C3 and C4 pseudoradical centers, integrating 0.31 and 0.93 e, respectively, just before the event.
6. The formation of the second C3-C4 single bond begins while the first O1-C5 single bond has reached 81% of its final population. Therefore, the mechanism of 32CA of nitrone **1** and nitroalkene **2a** proceeds by a two-stage one-step mechanism [34].
7. Both bond formations follow previously presented models [35,36], but show reverse regioselectivity to the one deduced from the Parr function.

## 3. Materials and Methods

### 3.1. Materials

*Z*-C-(3-pyridyl)-*N*-methylnitrone (**1**) was prepared via condensation of a 3-pyridyl aldehyde with the methylhydroxylamine in an etanolic solution according to the already known procedure [37]. Conjugated nitroalkenes were prepared from respective nitroalcohols via respective nitroalkyl esters according to protocols described in the literature [38,39,40]. Commercially available (Sigma-Aldrich Poland, Merck Poland) reagents and solvents were used in all experiments.

### 3.2. Analytical Techniques

HPLC analyses were carried out using a Knauer device with a UV VIS detector (LiChro-spher 18-RP 5 µm column, eluent: 75% methanol). M.p. values were measured on the Boetius apparatus and were uncorrected. IR spectra were derived from the FTS Nicolet IS 10 spectrophotometer. UV/Vis spectra were recorded using a spectrometer UV-5100 BIO-SENS and were determined for the 200–500 nm range. MS spectrums were registered using a high-performance liquid chromatograph Agilent 1200 conjugated with a spectrometer mas Agilent 6120 Agilent Technologies, Inc., Santa Clara, CA, USA. Stationary flow 0.35 mL/min acetonitrile/water = 50/50, column ReproSil-Pur Basic-C18, 3 µm, 100 × 2 mm (Dr. Maisch, High Performance LC GmbH, Beim Brückle 14, Ammerbuch, Germany). The isotopic pattern and high-resolution mass were calculated in the software package Perkin Elmer ChemDraw Prime v.20 (PerkinElmer Waltham, MA, USA). NMR spectrums were registered using Bruker AV500 (1H 500 MHz) spectrometers (Bruker; Billerica, MA, USA). All spectra were obtained in CDCl3 or DMSO-d6 solutions and the chemical shifts (δ) are expressed in ppm using the internal reference to TMS.

### 3.3. Cycloaddition between *Z*-C-(3-pyridyl)-*N*-methylnitrone and Nitroalkenes–General Procedure

A solution of nitroalkene (0.02 mol) and nitrone (0.01 mol) in dry benzene (25 mL) was mixed at room temperature for 24 h. The post-reaction mixture was filtered, and the solvent was evaporated in a rotary evaporator. The residue was recrystallized from the ethanol. Pure products were identified on the basis of spectral data. The obtained characteristics are collected in the Supplementary Materials.

### 3.4. ELF Computational Study

DFT calculations were conducted using the hybrid ωB97X-D functional [41], which is included in the GAUSSIAN 16 package [42], along with the 6-311G(d) basis set. All stationary points were characterized by frequency analysis; transition states (TSs) showed only one negative eigenvalue in their Hessian matrices; substrates and products showed only positive eigenvalues in their Hessian matrices. The influence of the solvent on the reaction was included by using the IEFPCM algorithm [43]. All calculations were made for molecules at 298.15 K and 1 atm.

The global electron density transfer [35,44] (GEDT) was calculated using the equation GEDT (f)n = ∑_(q∈f)_ q, where q are the charges, computed by natural population analysis [45,46] (NPA), of all atoms belonging to one of the two frameworks (f) at the TS. The global reactivity indices (electronic potential μ, chemical hardness η, global electrophilicity ω, and global nucleophilicity N) were calculated at B3LYP/6-31G(d) computational level using the equation described in [47].

Electron Localization Function (ELF) [46] analysis was performed using the TopMod package [47] at the standard cubical grid step size of 0.1 Bohr. To visualize the molecular geometries and ELF basin attractors, the GaussView program [48] was used.

## 4. Conclusions

[3 + 2] Cycloaddition reactions *Z*-C-(3-pyridyl)-*N*-methylnitrone with *E*-2-R-nitroethenes are realized under mild conditions and with full regio- and stereochemical control. Obtained products can be considered as nicotinoids, with possible applications as biological active compounds. The comprehensive MEDT study exhibits all important mechanistic backgrounds of the described transformations. In particular, according to the Domingo terminology, considered processes should be classified as two-stage, one-step processes.

## Figures and Tables

**Table 1:** The global reactivity descriptors calculated at the B3LYP/6-31G(d) level of theory. The electronic chemical potential μ, chemical hardness η, electrophilicity ω, and nucleophilicity N of studied reagents.

	μ	η	ω	N
3,3,3-trichloro-1-nitropropene **2a**	−5.74	5.35	3.07	0.69
3,3,3-tribromo-1-nitropropene **2b**	−5.50	5.03	3.00	1.09
1-nitropropene **2c**	−5.00	5.59	2.23	1.32
N-methyl-C-3-pyridylnitrone **1**	−3.65	4.33	1.53	3.29

## Data Availability

Not applicable.

## Supplementary Materials

The following supporting information can be downloaded at: https://www.mdpi.com/article/10.3390/molecules28083535/s1, Table S1. ELF valence basins populations, distances of the forming bonds, relative electronic energies, GEDT and IRC values of the IRC structures, MC-P11, defining twelve phases characterizing the molecular mechanism of the [3 + 2] cycloaddition reaction of nitrone **1** and 3,3,3-trichloro-1-nitropropene **2a**. Distances are given in angstroms, Å, GEDT values, and electron populations in an average number of electrons, e, relative energies in kcal mol^−1^ and IRC values in a.

## Sample Availability

Samples of the compounds are not available from the authors.
