# Supplementary material for: Full Regio- and Stereoselective Protocol for the Synthesis of New Nicotinoids via Cycloaddition Processes with the Participation of Trans-Substituted Nitroethenes: Comprehensive Experimental and MEDT Study
Source: Molecules. 2023 Apr 17;28(8):3535. doi: 10.3390/molecules28083535 (PMC10142438; doi:10.3390/molecules28083535)
Supplement: Supplementary file 1 [file molecules-28-03535-s001.zip › molecules-2331237-supplementary.pdf]

Supplementary Material

Full Regio- and Stereoselective Protocol for the Synthesis of New Nicotinoids via Cycloaddition Processes with the Participation of Trans-Substituted Nitroethenes: Comprehensive Experimental and MEDT Study

Jowita Kras, Przemysław Woliński, Roman Nagatsky,
Oleg M. Demchuk and Radomir Jasiński *

Contests:

[1] Synthetic procedures 2

[2] Physical characteristics of adducts . . 3

[3] Quantum-chemical calculations . . . 7

[1] Synthetic procedures

[1.1] Z-C-(3-pyridyl)-N-methylnitrone (1)

A solution of N-methyl-N-hydroxylamine hydrochloride (29.2 mmol), anhydrous sodium sulfate (14.1 mmol), and 3-pyridyl aldehyde (18.7 mmol), in ethanol (20 mL) was mixed at room temperature for 24 h. Sodium bicarbonate (85,71 mmol) was gradually added during stirring. The postreaction mixture was filtered. Anhydrous magnesium sulfate was added to the filtrate and filtered again. The solvent was evaporated. The isolation of the reaction product from the post-reaction mixture was performed via crystallization from ethanol. The pure product was identified on the basis of spectral data.

[1.2] Nitroalkenes

Conjugated nitroalkenes were prepared by pyrolysis of respective nitroalkyl carboxylates according to known procedures [Central European Journal of Chemistry, **12**, 586-593 (2014)].

[1.3] [3+2] Cycloaddition between Z-C-(3-pyridyl)-N-methylnitrone and nitroalkenes: general procedure

A solution of nitroalkene (0.02 mol) and nitrone (0.01 mol) in dry benzene (25 mL) was mixed at room temperature for 24 h (for the reaction between nitrone and 1-nitro-1-propene at 80°). The post-reaction mixture was filtered, and the solvent was evaporated in a vacuo. The residue was recrystallized from the ethanol. Pure products were identified on the basis of spectral data.

[2] Physical characteristics of adducts

***[2.1] 3,4-cis-4,5-trans-2-methyl-3-(3-pyridyl)-4-nitro-5-trichloromethyl-1,2-oxazolidine* 4a**

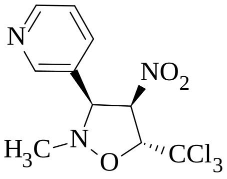

$C_{10}H_{10}N_3O_3Cl_3$; Pale yellow crystal needles; m.p.154°C from ethanol

MS (M+1) [m/z]: (325.9866 (100.0%), 327.9836 (95.9%), 329.9807 (30.6%), 326.9900 (10.8%), 328.9870 (10.4%), 330.9841 (3.3%), 331.9777 (3.3%), 326.9836 (1.1%), 328.9807 (1.1%).

IR [cm^{-1}]: 1560 i **1372** (-NO_2); **1102** (-C-N); **708** (C-Cl); **1027** (-N-O); **2961** (C_{sp3}-H); **1080** (C-$O_{(\delta)}$); **1427** (C-$O_{(\nu)}$)

^{1}H NMR, ppm [J:Hz] (500 MHz, $CDCl_3$): δ **2.74** (s, 1H, C$\underline{H}_3$); **4.19** (d, 1H, C$\underline{H}$-CCl_3, J=7,79); **5.49** (dd, 1H, C$\underline{H}$-NO_2, J=4,47, J=7,79); **5.54** (d, 1H, C$\underline{H}$-$C_{(Py)}$, J=4,47); **7.33** (dd, 1H, C$\underline{H}_{(Py)}$); **7.65** (d, 1H, C$\underline{H}_{(Py)}$); **8.59** (s, 1H, C$\underline{H}_{(Py)}$); **8.65** (d, 1H, C$\underline{H}_{(Py)}$);

^{13}C NMR [ppm] (125 MHz, $CDCl_3$): δ **43,19** ($\underline{C}H_3$); **74,33** ($\underline{C}$H-NO_2); **88,77** ($\underline{C}$H-CCl3); **94,58** ($\underline{C}Cl_3$); **94,93** ($\underline{C}$H-Py); **124,03** ($\underline{C}H_{(Py)}$); **127,13** ($\underline{C}_{(Py)}$); **135,07** ($\underline{C}H_{(Py)}$); **149,70** ($\underline{C}H_{(Py)}$); **151,32** ($\underline{C}H_{(Py)}$)

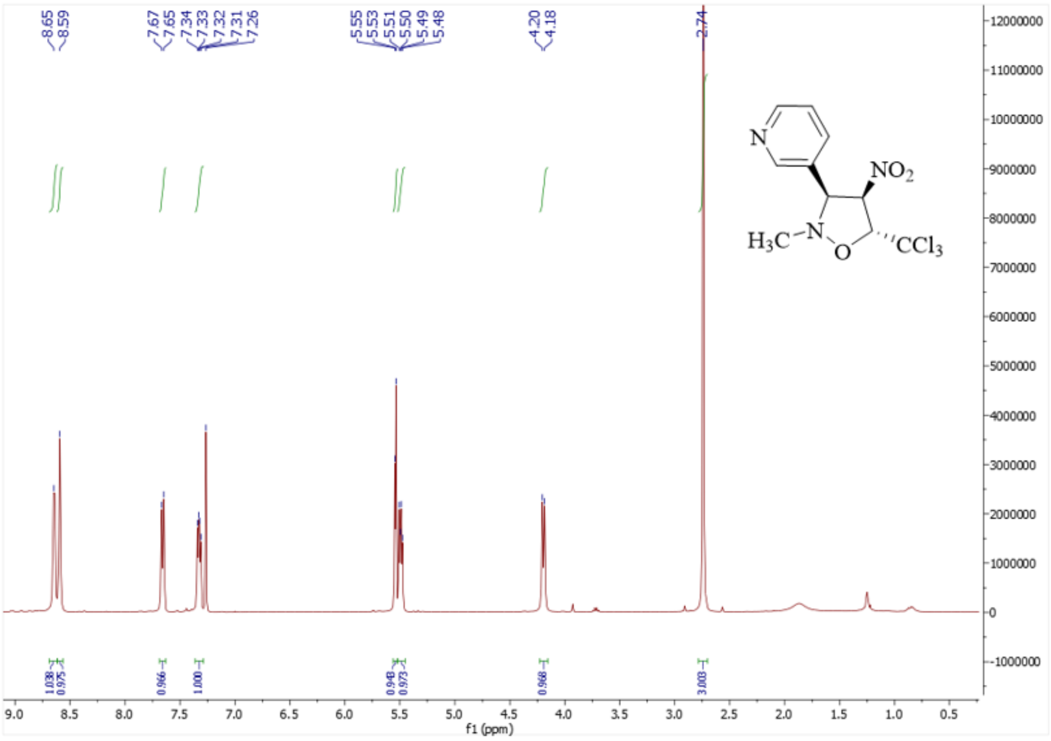
8.65
8.59
7.67
7.65
7.34
7.33
7.32
7.31
7.26
5.55
5.53
5.51
5.50
5.49
5.48
4.20
4.18
2.74
N
NO2
H3C
N
O
CCl3
f1 (ppm)

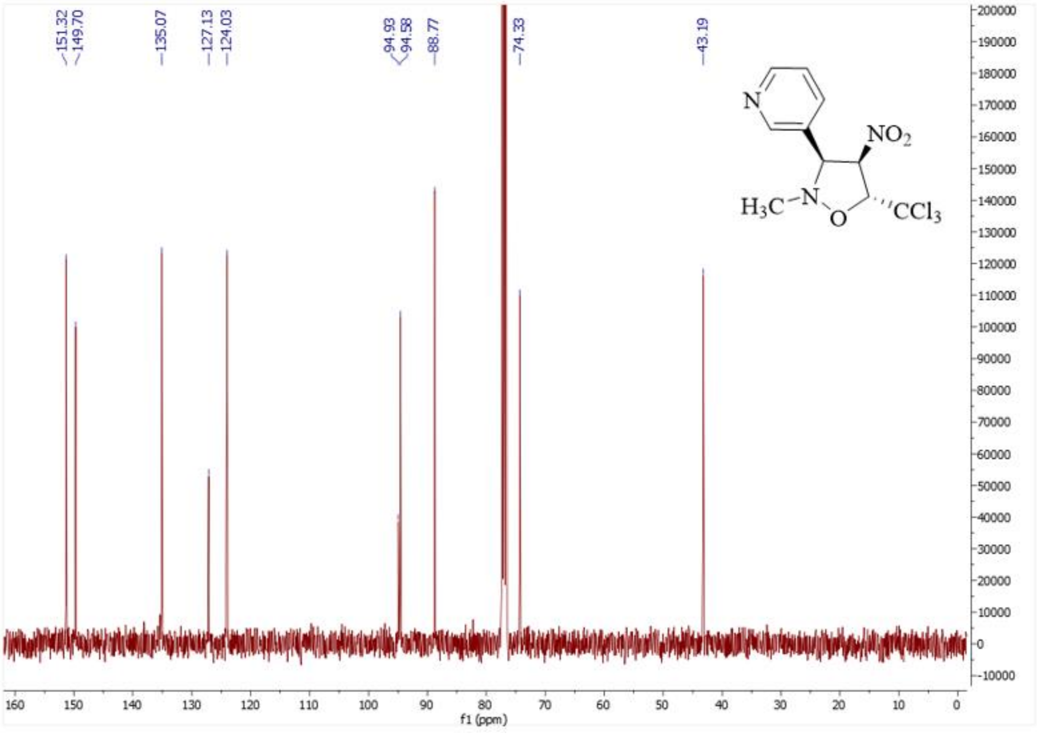
151.32
149.70
135.07
127.13
124.03
94.93
94.58
88.77
74.33
43.19
N
NO2
H3C
N
O
CCl3
f1 (ppm)

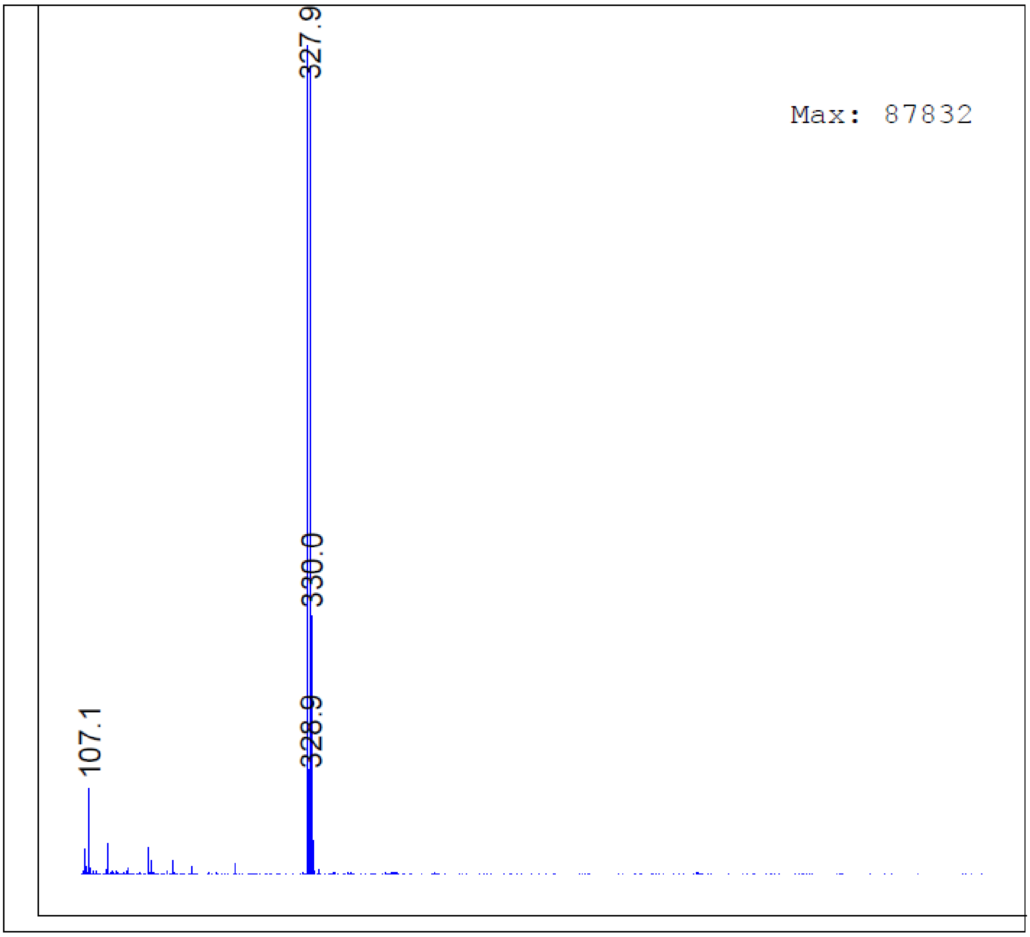

***[2.2] 3,4-cis-4,5-trans-2-methyl-3-(3-pyridyl)-4-nitro-5-tribromomethyl-1,2-oxazolidine* 4b**

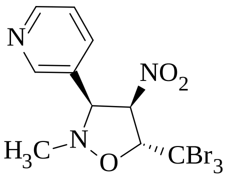

$C_{10}H_{10}N_3O_3Br_3$; Pale yellow crystal needles; m.p.168°C from ethanol

MS (M+1) [m/z]: (459.8330 (100.0%), 461.8310 (97.3%), 457.8351 (34.3%), 463.8289 (31.5%), 460.8364 (10.8%), 462.8343 (10.5%), 458.8384 (3.7%), 464.8323 (3.4%), 460.8300 (1.1%), 462.8280 (1.1%); m/z: 224.1035 (100.0%), 225.1069 (10.8%), 225.1006 (1.1%))

IR [cm^{-1}]: 1559 i **1365** (-NO_2); **1100** (-C-N); **547** (C-Br); **993** (-N-O); **2963** (C_{sp3}-H); **1069** (C-$O_{(\delta)}$); **1425** (C-$O_{(\nu)}$)

^{1}H NMR, ppm [J:Hz] (500 MHz, $CDCl_3$): δ **2.75** (s, 1H, $C\underline{H}_3$); **4.27** (d, 1H, $C\underline{H}$-CBr_3, J=8,07); **5.40** (dd, 1H, $C\underline{H}$-NO_2, J=4,51, J=8,07); **5.54** (d, 1H, $C\underline{H}$-$C_{(Py)}$, J=4,51); **7.33** (dd, 1H, $C\underline{H}_{(Py)}$); **7.67** (d, 1H, $C\underline{H}_{(Py)}$); **8.60** (s, 1H, $C\underline{H}_{(Py)}$); **8.65** (d, 1H, $C\underline{H}_{(Py)}$)

^{13}C NMR [ppm] (125 MHz, $CDCl_3$) δ**:** **36,89** ($\underline{C}Br_3$); **43,29** ($\underline{C}H_3$); **74,62** ($\underline{C}$H-NO_2); **90,02** ($\underline{C}$-CBr_3); **95,72** ($\underline{C}$H-Py); **124,06** ($\underline{C}H_{(Py)}$); **127,40** ($\underline{C}_{(Py)}$); **135,05** ($\underline{C}H_{(Py)}$); **149,70** ($\underline{C}H_{(Py)}$); **151,29** ($\underline{C}H_{(Py)}$)

***[2.3] 3,4-cis-4,5-trans-2-methyl-3-(3-pyridyl)-4-nitro-5-methylo-1,2-oxazolidine* 4c**

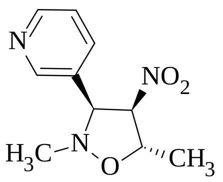

$C_{10}H_{13}N_3O_3$; Pale yellow semisolid

MS (M+1) [m/z]: 224.1

IR [cm^{-1}]: 1549 i **1372** (-NO_2); **1104** (-C-N); **2979** (C_{sp3}-H); **1054** (C-$O_{(\delta)}$); **1428** (C-$O_{(\nu)}$)

^{1}H NMR, ppm [J:Hz] (500 MHz, $CDCl_3$): δ **1,38** (d, 3H, C-$C\underline{H}_3$, J=6,31); **2.49** (s, 3H, N-$C\underline{H}_3$); **4.21** (d, 1H, $C\underline{H}$-C_{3Py}, J=8,09); **4.74** (dq, 1H, $C\underline{H}$-CH_3, J=6,31, J=5,23); **5.46** (dd, 1H, $C\underline{H}$-NO_2, J=5,23, J=8,09); **7.88** (d, 1H, $H_{(Py)}$); **7.37** (dd, 1H, $H_{(Py)}$); **8.53** (d, 1H, $H_{(Py)}$); **8.63** (s, 1H, $H_{(Py)}$)

^{13}C NMR [ppm] (125 MHz, $CDCl_3$): δ **17,28** (C-$\underline{C}H_3$); **42,55** (N-$\underline{C}H_3$); **97,03** ($\underline{C}$H-NO_2); **76,09** ($\underline{C}$-CH_3); **74,94** ($\underline{C}$H-Py); **123,56** ($\underline{C}H_{(Py)}$); **135,96** ($\underline{C}_{(Py)}$); **135,13** ($\underline{C}H_{(Py)}$); **149,43** ($\underline{C}H_{(Py)}$); **149,72** ($\underline{C}H_{(Py)}$)

[3] Quantum-chemical calculations

Cartesian coordinates are in Å

3,3,3-trichloro-1-nitropropene **2a**

E = -1701.175501 a. u.

C	1.46397000	-0.43850900	0.00010600
C	0.44756100	0.40309900	-0.00021800
H	1.43151400	-1.51698500	0.00044000
H	0.63238600	1.47188500	-0.00052800
N	2.83199600	0.08476600	-0.00003700
O	3.71055600	-0.75352200	-0.00005700
O	3.00391500	1.28574500	-0.00012100
C	-0.99918300	0.01834700	-0.00009000
Cl	-1.75108600	0.73223900	1.45886000
Cl	-1.75129600	0.73134000	-1.45919100
Cl	-1.26689600	-1.74026600	0.00050700

C-3-Pirydyl-N-methylnitone **1**

E = -456.159917 a. u.

C	0.32120000	-0.29401300	-0.00000300
C	0.76990000	1.03247900	-0.00000700
C	2.13742500	1.26835400	-0.00001000
C	3.01214500	0.19192100	-0.00001000
C	1.30050500	-1.30074100	-0.00000400
H	0.05328000	1.84023200	-0.00001200
H	2.52138300	2.28198500	-0.00000900

H	4.08710800	0.34943500	-0.00001200
H	0.99645400	-2.34618300	-0.00000400
C	-1.06431000	-0.72112000	0.00001300
H	-1.27576100	-1.78163100	0.00002600
C	-3.46027900	-0.51213700	0.00002200
H	-3.96888200	-0.14116900	0.88815200
H	-3.96885500	-0.14128200	-0.88817100
H	-3.42863400	-1.59965300	0.00009100
N	-2.10601600	0.06642500	0.00001200
O	-2.07771900	1.33172200	-0.00001000
N	2.60689400	-1.08127700	-0.00001000

TS

E = -2157.324987 a. u.

C	-2.34176000	-0.76359000	0.00367900
C	-2.29902800	-0.54229300	1.38491700
C	-3.37399200	0.08655400	1.98181600
C	-4.43932900	0.49690500	1.18631700
C	-3.46826500	-0.31498100	-0.69648100
H	-1.44389400	-0.85995700	1.96674500
H	-3.38596900	0.27369100	3.04860900
H	-5.29014500	1.00703500	1.62869900
H	-3.53605400	-0.46816400	-1.77094200
C	-1.32505200	-1.46021900	-0.76239300
H	-1.62258600	-1.83536200	-1.73685000
C	0.59716200	-2.86885600	-1.13580100
H	-0.03309200	-3.65969300	-1.53830900

H	1.05689300	-2.30056600	-1.94728000
H	1.37054300	-3.29840400	-0.50527400
N	-0.21243800	-1.98839400	-0.29947200
O	0.42047100	-1.35511600	0.66778000
C	0.18595700	0.52020600	-1.08825500
C	0.78776800	0.25677600	0.15534600
H	0.63522600	0.30719700	-2.04590000
H	0.32942100	0.81162400	0.96810200
N	-0.86040200	1.46417300	-1.16289100
O	-1.32842700	1.69592400	-2.27505500
O	-1.28926800	1.98311500	-0.13484400
C	2.30150700	0.30189400	0.30321400
Cl	2.78589100	2.02344800	0.16884800
Cl	2.80855300	-0.29330300	1.90989000
Cl	3.15453100	-0.62935800	-0.95988300
N	-4.49643800	0.30185900	-0.12982800

3,4-cis-4,5-trans-2-methyl-3-(3-pyridyl)-4-nitro-5-trichloromethyl-1,2-oxazolidine **4a**

E = -2157.383201 a. u.

C	2.33092300	0.48985000	-0.20921000
C	2.47357500	0.98141100	1.08451500
C	3.64114500	0.70151900	1.77604900
C	4.62375800	-0.05505100	1.14854100
C	3.38266000	-0.24853600	-0.74562600
H	1.68795500	1.57896700	1.53270700
H	3.79260300	1.06544300	2.78543900
H	5.55019500	-0.29227400	1.66413100

H	3.31548000	-0.63740100	-1.75998300
C	1.08875900	0.73473400	-1.03536100
H	1.36198100	0.81927700	-2.08811000
C	-0.37226800	2.57632100	-1.70501400
H	-0.98375800	1.88762500	-2.30008200
H	-1.01686500	3.33433900	-1.26395600
H	0.34904900	3.07173500	-2.35678700
N	0.38202700	1.94141900	-0.63238700
O	-0.51212200	1.52792900	0.41191900
C	-0.00511200	-0.36372200	-0.87289000
C	-0.75966300	0.13412700	0.35137300
H	-0.62172800	-0.43014800	-1.76655600
H	-0.34996900	-0.34151000	1.24660700
N	0.61268600	-1.72036100	-0.72288900
O	0.90157900	-2.27130400	-1.76434100
O	0.83497300	-2.14780500	0.38616200
C	-2.27622100	-0.14401600	0.38052800
Cl	-2.52686700	-1.91231600	0.34251500
Cl	-2.94021100	0.51306700	1.89761200
Cl	-3.13146500	0.59585100	-0.99669400
N	4.50950200	-0.52229700	-0.09316700

….

Structures	1	2a	MC	P1	P2	P3	P4	P5	P6	P7 TS	P8	P9	P10	P11	PR
Phases				I	II	III	IV	V	VI	VII	VIII	IX	X	XI	XII
d1(C10-O8)			3.060	2.303	2.142	2.078	1.877	1.850	1.789	1.730	1.658	1.627	1.533	1.459	1.416
d2(C9-C5)			3.323	2.929	2.816	2.774	2.637	2.617	2.567	2.511	2.421	2.372	2.143	1.793	1.558
IRC			-14.75	-4.32	-2.89	-2.40	-1.02	-0.84	-0.42	0.00	0.60	0.90	2.16	3.96	
GEDT			0.02	0.12	0.17	0.19	0.24	0.24	0.25	0.25	0.22	0.20	0.07	-0.12	
dE			-3.4	3.7	6.6	7.7	11.0	11.4	11.9	12.1	11.5	10.8	4.5	-12.2	-30.0
V(O1)	2.91		2.92	2.87	2.88	2.88	2.91	2.92	2.97	2.80	2.59	2.60	2.54	2.53	2.47
V'(O1)	2.99		3.02	3.02	3.00	2.97	2.91	2.90	2.86	3.22	2.72	2.69	2.59	2.52	2.57
V(N2,O1)	1.44		1.43	1.38	1.35	1.34	1.26	1.24	1.21	1.18	1.14	1.12	1.05	1.00	0.94
V(C3,N2)	3.78		3.74	3.77	3.79	3.81	3.92	3.05	2.90	2.79	2.66	2.50	2.22	1.96	1.84
V(C6,N2)	2.00		2.00	1.99	1.99	1.98	1.94	1.93	1.92	1.89	1.88	1.86	1.87	1.88	1.87
V(C7,C3)	2.34		2.37	2.38	2.38	2.38	2.40	2.40	2.41	2.41	2.42	2.40	2.28	2.15	2.09
V(C7,C8)	2.82		2.79	2.81	2.81	2.81	2.82	2.82	2.83	2.83	2.83	2.84	2.88	2.94	2.94
V(C7,C12)	2.71		2.70	2.69	2.68	2.68	2.67	2.67	2.66	2.66	2.66	2.66	2.68	2.71	2.76
V(C4,C5)		1.75	1.75	1.78	1.78	3.48	3.09	3.07	2.88	2.71	2.53	2.45	2.26	2.10	2.03
V'(C4,C5)		1.80	1.77	1.71	1.72										
V(C4,N13)		2.28	2.32	2.44	2.52	2.58	2.62	2.63	2.64	2.65	2.65	2.62	2.57	2.25	2.22
V(N13)		0.18	0.17	0.06										0.28	0.22
V'(N13)		0.18	0.03												
V(O1,C5)											0.76	0.80	1.05	1.21	1.29
V(C3,C4)													1.26	1.64	1.88
V(N2)								0.90	1.10	1.28	1.49	1.60	1.92	2.20	2.31
V(C3)												0.12			
V(C4)							0.44	0.47	0.56	0.63	0.73	0.78			
V(C5)									0.07						

Table S1. ELF valence basins populations, distances of the forming bonds, relative electronic energies, GEDT and IRC values of the IRC structures, MC-P11, defining twelve phases characterizing the molecular mechanism of the [3+2] cycloaddition reaction of nitrone 1 and 3,3,3-trichloro-1-nitropropene 2a. Distances are given in angstroms, Å, GEDT values, and electron populations in an average number of electrons, e, relative energies in kcal mol^{-1} and IRC values in a.
